# Report of a Case of Rupture of the Uterus, Presented to the Medical Association of Georgia

**Published:** 1860-05

**Authors:** S. W. Burney

**Affiliations:** Forsyth, Ga.


### ARTICLE II.

*Report of a Case of Rupture of the Uterus, presented to the Medical Association of Georgia.* By S. W. BURNEY, M. D., Forsyth, Ga.

Laceration of the Uterine Viscus may occur in any case of preternatural or dystocial labor. During an obstetrical practice of thirty years, however, embracing twelve or fifteen hundred cases of labor, I have encountered but a single case of rupture of the Uterus. And among those of my cotemporaries of equal experience, with whom I have had an interchange of opinions in so far as the occurrence of remarkable cases are concerned, I have yet to see the first practitioner whose misfortune it has been to encounter an injury of this kind; it must, therefore, be of rare occurrence. I have concluded to call the attention of the Medical Association of Georgia, to the following case, on account of a novel fact connected with it, rather than to any benefits that may accrue to suffering humanity in consequence thereof. Whenever a solution of continuity of the muscular substance of the womb takes place, the friends and relatives of the unfortunate subject become terror stricken; and the attending Accouchier, unless he be endowed with more than a usual quantity of moral courage, loses, as well, his nerve as his presence of mind, and is, therefore, disqualified for the proper discharge of the duties imposed upon him. In this way, reputations are won and lost all over the world.

This accident, the most formidable of all those which may happen, either during pregnancy or labor, admits, according to all writers, of two order of causes—one predisposing and the other exciting or immediate. With those cases occurring during uterine gestation, we have nothing to do on this occasion, because the case under consideration was one of a different character. Among the predisposing causes, the first rank is occupied by all those obstacles, which, at the moment of delivery, may prevent the advance of the fœtus, and compel the uterus to the execution of prolonged and powerful contractions, such as deformity of the pelvis, strongly marked obliquities of the uterus, scirrhus, fibrous, and other tumors, cartilaginous hardness and obliteration of its orifice, occlusion of the vulva and vagina, abnormal positions, and some monstrosities of the fœtus. Another category of predisposing causes, includes the weakening of the uterine parietes, either from blows, falls, or other external violence, which have occasioned the injury—inflammation, softening, or ulceration of some part of the organs, or from frequent pregnancies at short intervals. Lastly, a third embraces the malposition the woman may assume in her efforts during labor, the improper direction she may give these efforts, sneezing, fits of coughing, vomiting, bursts of anger and convulsions. In a word, among the predisposing causes, we must include, on the one hand, those circumstances capable of producing a partial weakening of the uterine parietes, and, on the other, all those which cause the uterus to meet, during its contraction, with a resistance too great for its powers to overcome. Now, as none of these predisposing causes obtained in our case, we dismiss them without a word of comment.

The immediate cause was ascribed by a majority of old writers, to the violent movement of the fœtus; but in this respect, it would appear they had mistaken the effect for the cause, and that the movements felt by the woman, at the time of the occurrence of the rupture, were performed by the fœtus only after it had passed into the abdominal cavity. We must, therefore, seek elsewhere, the true, efficient cause of rupture. During pregnancy, there can hardly be any other than compressions exerted on the uterus, either mediately, as might happen to a pregnant woman squeezed between a wall and a vehicle; or immediately, by the sudden contraction of the abdominal muscles. In these two cases, in fact, the uterus thrust against the vertebral colemn, which does not yield, and supported by the anterior parities of the belly, converted by its contraction into an almost solid plane, must yield and rupture at its sides, where it is not supported. During labor by the action of the voluntary muscles, if the child sometimes becomes the instrument of laceration, it is never actively, but, as is remarked by Bandelocque, its agency does not differ from that of any inert solid body, with an angular surface, upon which the uterus might violently counteract. The force necessary to produce the rupture, need not always be very great; it suffices that in each given case it should be superior to the resistance opposed by the point predisposed to laceration. Many other causes of laceration of the uterus might be mentioned, but those now enumerated are sufficient for our purpose.

As brevity is always desirable in the performance of a duty, like that one I have voluntarily imposed upon myself, I shall at once, without farther trespassing upon your patience, proceed to the discharge of the task I have more immediately in view.

On Thursday, the 22d of last month, I was invited, early in the morning, by Mr. A. Lelner, of Monroe county, to make a visit to a negress of his in labor with her eighth child. The practitioner already in attendance requested me, through Mr. L., to bring with me a hook and perforator. Two and a half hours after the message was dispatched after me, I reached the patient's bed side. She was complaining of an acute pain in the precordial region, with general soreness over the whole abdominal region—extremities cold, pulse extinct, fainting fits frequent, the patient all the time saying she should suffocate unless she were allowed to get out of bed and stand on her feet. Her mind was perfectly lucid and as calm as could be reasonably expected under the circumstances. I at once touched the womb, without being able to find any part of the child presenting. I, however, found the placenta attached to the umbilical cord lying betwixt her thighs. The placenta was at once separated from the cord, and making a conductor thereof, my hand properly lubricated was carried as far into the cavity of the womb as was allowable from its contracted condition. Failing to find any part of the fœtus in utero, I at once became suspicious of the true state of the case. I learned from the practitioner present that he had been called to the patient on the Tuesday preceding. The first evidence of approaching labor was the discharge of an unusual quantity of liquor amnii. The pains during the first stage were slow and inefficient, though very cutting. No bearing down effort was evinced until 12 o'clock Wednesday night, soon after which the head of the fœtus engaged in the superior strait of the pelvis. At or about 4 o'clock, A. M., the head had entered fully the cavity of the bones, and he was encouraged to believe that a few more pains would accomplish the result. The woman now suddenly cried out, "I feel just like a butcher knife was being driven through my body." This pain commenced at the precordial region, and subtended to the back. She also complained heavily of a feeling of suffocation, and insisted on getting out of bed to prevent this catastrophe. This she did, as the doctor assured me, albeit he and three other adult persons used all their powers to keep her on the bed. So soon as her feet struck the floor the blood began to run from her in a stream, and the doctor supposed she lost, first and last, at least three pints or half a gallon. Before they succeeded in getting the patient upon the bed, the placenta came away and hung by the cord at the vulva. There was no hemorrhage present when I examined, per vaginam, but she had not experienced a moment's ease in the region of the stomach from the time she was seized therewith, until I reached her bed-side, at 9 o'clock, A. M.

After learning these facts, I placed my right hand, for the first time, on the abdominal region, and plainly felt the head, arms and back of the fœtus through its parieties. As the most important indication was to bring about re-action and relieve the violence of the pain and oppression under which she had labored for five hours, 60 drops of Tinct. of Opii was at once given—hot toddy given in small portions every ten minutes, and mustard and other stimulating remedies were applied to the extremities. The pain not subsiding any in an hour, a large dose of sulph. morphine was given, and toddy and other appliances persevered in. In the course of two hours she became calmer, and I determined to make an effort to reach the feet of the child, and, if possible, to accomplish its delivery by manual efforts. I found this impossible, however, on account of the contraction of the uterus. I satisfied myself, however, that the rent was not in the "cul de sac" of the vagina. The question now arose, what could be done to save the life of the woman. The child was left out of the question, because it had obviously perished at the time the placenta was discharged. The opetion of gastrotomy in the then condition of the patient was not to be thought of, as she would have sank under the surgeon's knife. The plan determined upon, was to use all the means and appliances at hand to secure reaction, and, in the event we succeeded in accomplishing this desideratum, we would then determine as to whether the operation of gastrotomy would be allowable and proper. This, however, we failed in accomplishing, the patient gradually sinking, without either any return of pulse or heat to the extremities, dying at 6 o'clock Friday morning.

It is probably necessary and proper to say that the patient did not feel the least pain in the uterine region from the time the laceration took place, till death ended her sufferings. I would also say, for the benefit of those who are fond of abstractions, that notwithstanding the patient was strong and vigorous, all of her seven children previously born, came into the world without life or motion, and that only one out of the seven were saved by artificial respiration. This was not owing to any contraction of the pelvis, as I satisfied myself several years ago, when I attended her in labor with her fifth or sixth child. The labor was somewhat tedious, but I could see no reason then, nor do I now, why her child should have been still-born, and especially why my efforts then instituted should have failed to give life and energy to her infant. I submit facts, and leave it for others to speculate thereupon.

The most remarkable feature connected with this case was the discharge of the placenta at the time the laceration was supposed to have taken place. This phenomena could not have been owing to an implantation of the after-birth over or very near the os uteri. Had such been its position, hemorrhage would most certainly have taken place at, or shortly after, the pains of travail set in. Besides, the gentleman in attendance assured me he touched the woman sufficiently often to have detected this mal-position, had it been present. Then, we must look elsewhere for facts and reasoning for the solution of this strange problem. I can account for it only in this way. It is well known that so soon as the fœtus escapes from the uterine cavity it is the wont of this organ to contract upon its own walls—thus lessening its cavity and securing the expulsion of the placental mass. This happens in all cases terminating favorably. But for this tonic contraction, we should be compelled to intervene in every case of labor, to extract the after-birth.

Well, in the case now detailed, the vent must either have occurred at or very near the implantation of the placenta thus assisting nature in its expulsion ; or there was energy enough left in the uterine fibres, to secure its contraction, and thus discharge this mass. It is probably the only instance on record, and is, therefore, of sufficient importance to excuse me for having thus troubled the Association.

Some may feel and express surprise at my neglect to make in this case a post mortem examination. Such certainly was our wish, but the great repugnance of the patient's mother to have her daughter cut open', as she termed it, together with some little prejudice of the owner, to such examinations, especially as he could not see how one, in this case, could benefit suffering humanity, forced us reluctantly to forego this desideratum.

All of which is respectfully submitted.

